# The Molecular Organization of Self-awareness: Paralimbic Dopamine-GABA Interaction

**DOI:** 10.3389/fnsys.2020.00003

**Published:** 2020-01-28

**Authors:** Hans C. Lou, Kristine Rømer Thomsen, Jean-Pierre Changeux

**Affiliations:** ^1^Center of Functionally Integrative Neuroscience, Institute for Clinical Medicine, Aarhus University, Aarhus, Denmark; ^2^Department of Psychology and Behavioral Sciences, Center for Alcohol and Drug Research, School of Business and Social Sciences, Aarhus, Denmark; ^3^Institut Pasteur, Paris, France

**Keywords:** self-awareness, default mode network, conscious experience, GABA, dopamine, addiction

## Abstract

The electrophysiology of the paralimbic network (“default mode”) for self-awareness has drawn much attention in the past couple of decades. In contrast, knowledge of the molecular organization of conscious experience has only lately come into focus. We here review newer data on dopaminergic control of awareness in humans, particularly in self-awareness. These results implicate mainly dopaminergic neurotransmission and the control of GABAergic function directly in the paralimbic network. The findings are important for understanding addiction, developmental disorders, and dysfunctional consciousness.

## Introduction

Self-awareness is a conscious experience with the self as an object. It is an essential part of conscious experience of the world, and is a tool for conscious self-monitoring and for controlling behavior. Its default may have grave consequences. The “neural correlates” of self-awareness have been studied by several investigators, including Devue and Brédart (2011) and D’Argembeau (2013). Generally, a cortical, paralimbic network has been proposed as a correlate for self-awareness. It often included prefrontal and medial parietal regions, but it remained unknown whether these regions were indeed instrumental in that function.

## The Paralimbic Network: From “Correlations” to Being “Instrumental” in Self-awareness

Based on the effect of temporary dysfunction of brain regions induced by transcranial magnetic stimulation (TMS), we have realized that a paralimbic network is indeed instrumental in self-awareness: the network is not only *active* during self-awareness (Kjaer et al., 2002; Lou et al., 2004), but is also *causal* for that function (Lou et al., 2004; Luber et al., 2012). Its core cortical regions, medial prefrontal and medial parietal are interneuron-rich hubs with multiple connections (Freund, 2003). They are usually linked with activity in the angular gyri, insula, and subcortical regions including striatum (Slagter et al., 2017) and thalamus (Rømer Thomsen et al., 2013; Bachman and Hudetz, 2014). The network is often termed the “default mode network” because it defaults when attention is turned away from the self to other tasks (Lou et al., 2017). The medial prefrontal and medial parietal regions are “hubs” of the network. They interact *via* GABA-induced synchronized gamma oscillations (Rømer Thomsen et al., 2013; Joensson et al., 2015).

## Dopamine-GABA Interaction in Conscious Experience

**Figure 1:**
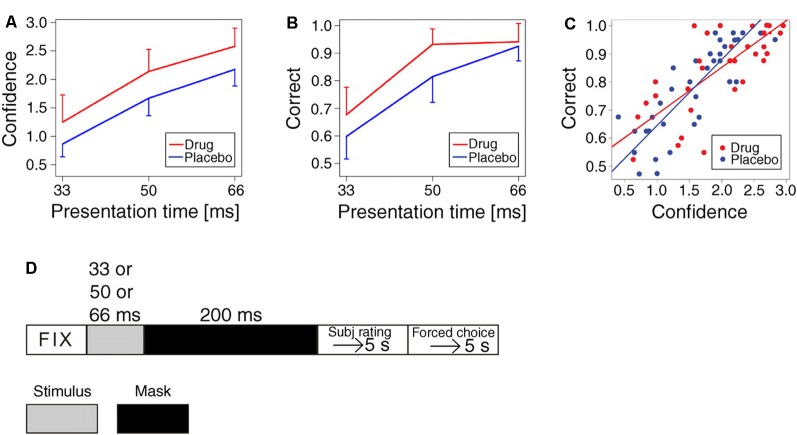
Effect of dopaminergic activation on confidence in seeing words and on accuracy in word recognition task. **(A)** Increase in the subjective rating of confidence (scale 0–3) for words presented at 33 ms, 50 ms, and 66 ms (means and standard deviations of means, *p* = 0.0018). **(B)** Increase in accuracy (percentage of correct responses, in word recognition task by forced choice, one distractor). The expectation from chance: 50%. Note ceiling effect (*p* = 0.006). **(C)** All observations of correct answers as a function of confidence, showing a significant effect (*p* < 0.0001, regression with random effect). The pergolide-treated group and the placebo groups had identical regression lines statistically. **(D)** Experimental timeline (Lou et al., 2011, p. 3). Adapted with permission from Lou et al. (2011).

Functional brain imaging has indicated that abnormal conscious experiences in schizophrenia, like hallucinations and delusions, are associated with abnormal dopaminergic neurotransmission (Changeux and Lou, 2011; Palmiter, 2011). In order to test if this association is causal, we used signal detection tasks and subsequent subjective interpretation, i.e., by setting a criterion for when the sensory signal-to-noise ratio provides confidence that a stimulus is present (Lou et al., 2011). We examined the effect of increasing dopamine activation and showed that dopaminergic stimulation with the D_1_ and D_2_ receptor agonist pergolide is effective in increasing confidence in seeing words and in improving performance in a forced-choice word recognition task. This demonstrates neurotransmitter regulation of subjective conscious experience of perception and provides the first direct evidence that dopamine is instrumental in conscious experience (Lou et al., 2011; Figure 1). Such activation depends on fast-spiking GABAergic interneurons (Changeux and Lou, 2011), which intermittently inhibit pyramidal cell activity to produce gamma oscillations necessary for effective neurotransmission (Freund, 2003; Lou et al., 2011; Rømer Thomsen et al., 2013; Joensson et al., 2015).

## Site of Dopamine-GABA Interaction

**Figure 2:**
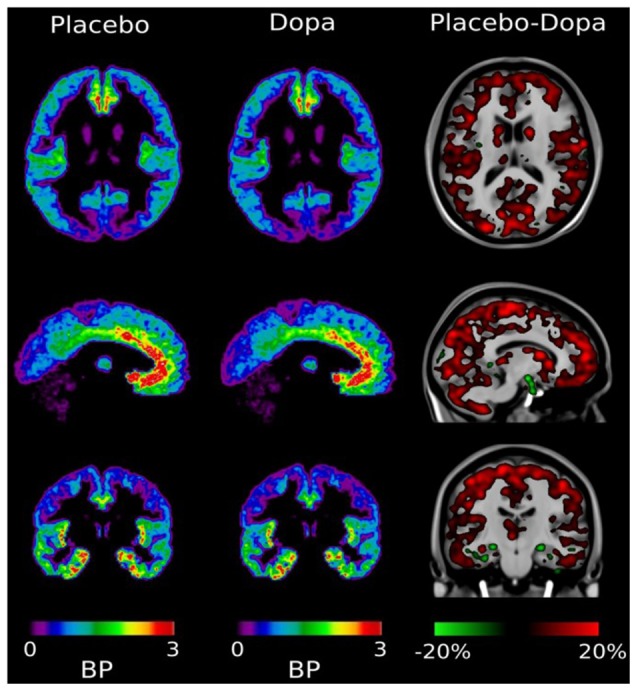
Distribution of [11C] Ro15-4513 GABA receptor ligand. After the placebo, the ligand was mainly bound in the medial-inferior prefrontal cingulate cortex, and right and left insula. After dopamine challenge ligand binding is reduced, with a general reduction of between 5% and 20% throughout the cortex. In hippocampal regions there were small foci of increased binding potential. Adapted with permission from Lou et al. (2016).

The site of dopamine-GABA interaction for self-awareness in the human brain was unknown until recently. To clarify this issue, we have used a PET ligand for GABA receptor binding. With this ligand, we were able to detect changes in dopamine-induced GABA binding under different physiological conditions at well-defined brain sites (Lou et al., 2016). It was found that GABA ligand binding was maximal in the medial anterior paralimbic region (cingulate gyrus), indicating empty GABA binding sites in these locations. Orally given dopamine reduced free GABA ligand binding sites throughout the cortex including the paralimbic system (Figure 2). In other words: dopamine increases GABA binding directly in the human paralimbic cortex, concomitantly with increased self-awareness and conscious experience (Lou et al., 2016). The effect is faltering in problem gambling, reflecting faltering self-monitoring and self-awareness (Møller et al., 2019). This development brings the properties of the GABA receptor molecule into focus.

## Functional Anatomy of GABA Receptors

The GABA receptors are constructed as ligand ion channels. According to Stephens et al. (2017), they are “organized as a pore between five protein complexes. The pore allows passage of negative chloride ions, and, hence, the generation of electrical pulses when stabilized in an open conformation. This stabilization occurs by binding of GABA to the complex. The binding is not specific for GABA. The affinity of other molecules, whether physiologic, including dopamine, and foreign molecules depends on the protein composition of the five pentameric molecules constituting the pore and determine the function of the synapse. The subtype composition of the pentameric pore is abnormal in addiction.” This may explain the abnormal interaction between dopamine and GABA receptors in gambling disorder, a prime example of dysfunctional self-monitoring and self-control (Møller et al., 2019). Related to this, a number of studies suggest that altered GABA neurotransmission plays an important role in substance addiction (Lingford-Hughes et al., 2012, 2016) or gambling (Mick et al., 2017).

## Self-awareness Network During Infancy and Childhood

In premature infants, functional MR imaging together with diffusion tensor imaging-based tractography has been used to study the relationship between performance on the Bayley Scales of Infant Development and early myelination (Cui et al., 2017). The Bayley Scales of Infant Development is a developmental play task that derives a developmental quotient (rather than an intelligence quotient). The authors confirmed such a link by showing that scores on the Bayley Scales of Infant Development were significantly associated with cingulate fractional anisotropy and radial diffusivity (Cui et al., 2017). This suggests that interconnecting axonal pathways within the default mode network are of critical importance already in the early neurocognitive development of infants.

Newborn infants already show a form of “basic consciousness” by establishing rudimentary eye contact with their mother (Lagercrantz and Changeux, 2009). A classical test for the presence of self-awareness in infants (and animals) is the mirror recognition test, where the infant is placed before a mirror with a spot marked on his/her forehead. A positive response is usually present at about 2 years of age. It requires the infant to try to remove the spot on his/her head, and not on the mirror (Anderson, 1984).

A recent review showed that the default mode network follows an inverse U-shape, where it is weaker in children and elderly and stronger in adults. Cognitive function is positively correlated with default mode network functional connectivity (Mak et al., 2017).

## Deficient Self-awareness and Pathology

In later childhood and adulthood, disturbance of the paralimbic network is linked to severe pathology. Thus, deficient GABA neurotransmission is prominent in disorders with poor self-awareness and self-monitoring such as addiction (Lingford-Hughes et al., 2012, 2016; Mick et al., 2017; Møller et al., 2019), autism (Hashemi et al., 2017), anosognosia, hallucinations, and delusions (Therriault et al., 2018), progressing to schizophrenia (Rikandi et al., 2018) major depression (Rikandi et al., 2018), and/or dementia (Lin et al., 2015). Even in the vegetative state (also termed unresponsive wakefulness syndrome, Laureys et al., 2010), recovery of the paralimbic network is tightly linked to clinical recovery (Thibaut et al., 2019). Finally, a large clinical study of the possible therapeutic effect of apomorphine, a dopaminergic drug, is now underway (Sanz et al., 2019).

To determine if deficient GABA neurotransmission in pathology could be a primary event or secondary to toxic or pathological effects in more complex disorders, we examined if deficient dopamine-GABA neurotransmission was present in a relatively mono-symptomatic disorder such as gambling disorder (Møller et al., 2019). This was indeed the case. Therefore, normal interaction between these transmitters in the medial paralimbic system seems to be fundamental for brain function.

## Dopamine Activity Linked With Other Transmitters

The basal forebrain part of the system is not only regulated by *dopamine*. It is also a prominent site of *cholinergic* activity. Interaction between the cholinergic and dopaminergic system *via* GABA receptors has been well described (Changeux and Lou, 2011; Takács et al., 2018). Thereby it promotes functions related to conscious experiences such as attention, learning and memory, and sleep-wake alternation (Lin et al., 2015).

## Vulnerability of Paralimbic Network

The widespread dysfunction of self-awareness in disease is likely to be a consequence of the exceedingly high oxygen demand of the paralimbic network. The high oxygen requirement is considered to be the result of dense concentrations of parvalbumin GABAergic interneurons in the richly connected hubs of the paralimbic network. In particular, the fast gamma oscillations are susceptible to metabolic disruptions because of their high energy-demand (Kann et al., 2016).

## Discussion

Until recently, conscious experience and self-awareness were considered off-limits for the natural sciences. Neurobiological research shunned the “hard question” of how conscious experience and self-awareness arise from a physical basis. Hence, it has been fashionable to limit neuroscience to try to identify neural “correlates” of conscious experience and self-awareness. The risk is evident for arriving at two parallel worlds: a mental and a physical, without understanding how they interact. This limitation has impeded our understanding of the biological function of self-awareness, and how it may account for disease. We have here reported data showing that self-awareness and conscious experience can be disturbed by electrophysiological manipulation of the paralimbic network (Lou et al., 2004; Luber et al., 2012). Therefore, we may conclude that the network is instrumental for these functions. Newer data reviewed here also strengthen the conclusion by showing that dopaminergic agents may stimulate conscious experience *via* GABA receptors in the paralimbic network. This finding has already resulted in a large and promising study of disabled persons with faltering self-awareness and consciousness (Sanz et al., 2019).

## Author Contributions

HL conceptualized the review and wrote the initial draft. KR and J-PC participated in writing the manuscript.

## Conflict of Interest

The authors declare that the research was conducted in the absence of any commercial or financial relationships that could be construed as a potential conflict of interest.
